# Dural Arteriovenous Fistula Presenting As Pulsatile Tinnitus

**DOI:** 10.5334/jbsr.2619

**Published:** 2021-11-09

**Authors:** Maxime Van Sumere, Luc Defreyne, Vincent VandeVyver

**Affiliations:** 1 Ghent University, BE; 2 Ghent University Hospital, BE; 3 AZ Alma, BE

**Keywords:** Dural arteriovenous fistula, pulsatile, tinnitus, interventional, radiology, embolization

## Abstract

**Teaching point**: We report magnetic resonance imaging characteristics of a dural arteriovenous fistula (dAVF), a possible cause of pulsatile tinnitus.

## Case study

A 39-year-old woman with pulsatile tinnitus was referred to the radiology department to evaluate a possible underlying (vascular) cause. Otological and neurological examinations were completely normal.

**Figure 1:**
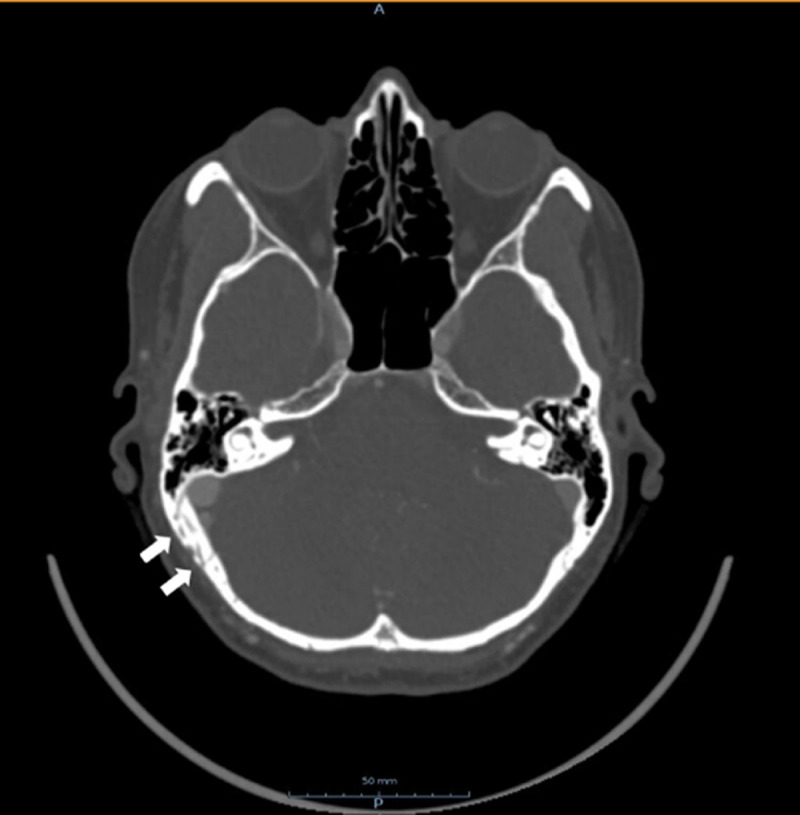


**Figure 2:**
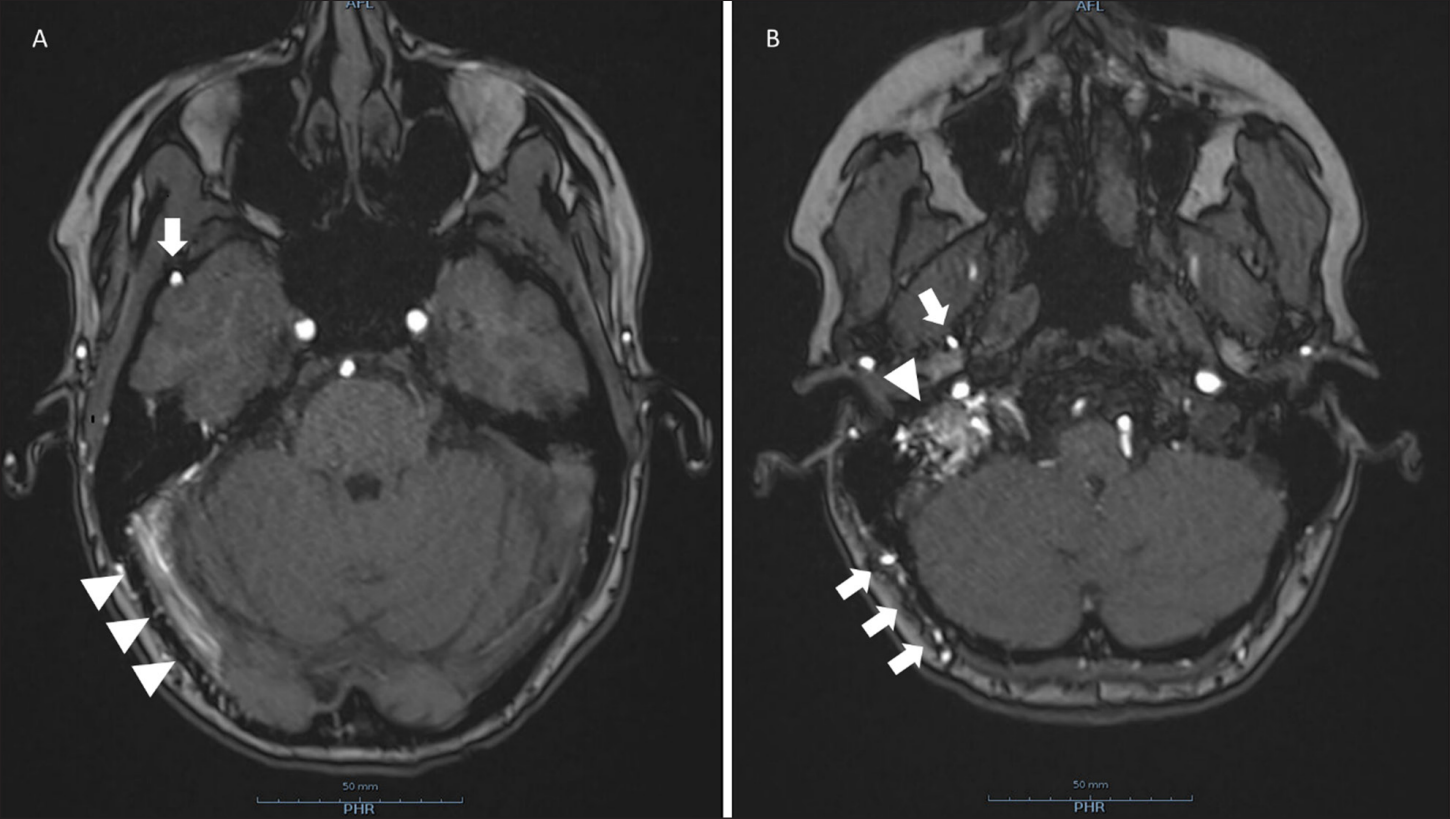


Doppler-ultrasonography of the carotids revealed turbulent flow in the right external carotid artery with increased flow velocities during diastole. Computed tomography angiography (CTA) of the carotids showed widened transosseous vessels at the level of the right occipital bone (***Figure 1***, arrows). The patient underwent magnetic resonance imaging (MRI). The non-contrast enhanced time-of-flight (TOF) images showed a dilated middle meningeal artery (***Figure 2A*** and ***2B***, single arrow) with widened transosseous vessels to the right occipital bone (***Figure 2B***, arrows). Flow voids can be seen in the right transverse and sigmoid sinus (***Figure 2A***, arrowheads) and internal jugular vein (***Figure 2B***, arrowhead), indicating arterial flow. There is a preserved antegrade flow in the dural sinuses, retrograde cortical venous drainage (RCVD) is absent. The diagnosis of a dural arteriovenous fistula (dAVF) (Cognard I) between the middle meningeal artery and the transverse and sigmoid sinuses was made. The patient was referred to an interventional radiologist. A wait-and-see policy was proposed, but at the patient’s request Onyx embolization of the dAVF was performed.

## Comment

DAVF is a rare, acquired vascular malformation with unknown aetiology involving arteriovenous shunts between dural arteries and dural sinuses. DAVF is usually seen in middle-aged to elderly patients. DAVF may remain asymptomatic or manifest as headache, diplopia, pulsatile tinnitus or intracranial haemorrhage. DAVFs are mostly situated in the transverse and sigmoid sinuses, with arterial feeding from branches of the external carotid artery. However, dAVF can also occur at the level of other dural sinuses with arterial feeding from branches of the internal carotid artery or vertebral arteries. DAVFs drain either directly into the dural sinuses or via cortical veins (RCVD).

If a dAVF is suspected, MRA without contrast enhancement should be performed, with TOF images being the most valuable sequence. In case of an inconclusive examination, or for extensive investigation of hemodynamics, selective angiography is necessary, which remains the gold standard.

MR(A) findings of a dAVF include one or more widened, tortuous feeding arteries with strong collateralisation, widened transosseous vessels and flow voids in the involved dural sinuses. Venous sinus thrombosis is often seen. In the presence of RCVD, dilated, tortuous cortical veins and white matter oedema due to venous congestion may be observed.

RCVD is the key feature to classify and treat dAVFs (Cognard classification). The presence of RCVD is associated with an increased risk of intracranial bleeding. When RCVD is absent (Cognard I and IIa), a wait-and-see policy is possible. In case of high-grade dAVFs (Cognard IIb-V), treatment is necessary. DAVF can be successfully treated with Onyx embolization [1].
